# Risk factors for venous thromboembolism in patients with diabetes undergoing joint arthroplasty

**DOI:** 10.1186/s12891-021-04453-9

**Published:** 2021-07-06

**Authors:** Wei Deng, Lili Huo, Qiang Yuan, Deyong Huang, Quan Li, Wei Tian

**Affiliations:** 1 Department of Endocrinology, Beijing Jishuitan Hospital, Beijing, China; 2 Department of Spine Surgery, Beijing Jishuitan Hospital, No. 31, Xinjiekou East Street, Xicheng District, 100035 Beijing, People’s Republic of China; 3 Department of Orthopedic, Beijing Jishuitan Hospital, Beijing, China

**Keywords:** Diabetes, Total knee arthroplasty, Total hip arthroplasty, Venous thromboembolism, Deep venous thrombosis

## Abstract

**Background:**

Venous thromboembolism (VTE) is a significant complication after joint arthroplasty. Diabetes is related to a few changes in coagulation and fibrinolysis that may lead to thrombophilia. We aimed to investigate the incidence of postoperative VTE and associated risk factors among patients with diabetes undergoing total hip (THA) or total knee anthroplasty (TKA) in a single centre in China.

**Methods:**

Patients with diabetes who underwent THA or TKA from January 2016 to December 2018 (*n* = 400) at Beijing Jishuitan Hospital were recruited in this study. Lower limb venous Doppler ultrasound was performed before and after surgery to confirm deep venous thrombosis (DVT). Computer tomography pulmonary angiography was done to confirm pulmonary embolism (PE) for those with new postoperative DVT and typical symptoms of PE. A multivariate logistic regression model was conducted to examine factors associated with the development of postoperative VTE.

**Results:**

The overall incidence of postoperative VTE in patients with diabetes after THA or TKA was 46.8 % (187 out of 400). Among the 187 VTE patients, 7.5 % (14 out of 187) had proximal vein thrombosis and 92.5 % (173 out of 187) had distal vein thrombosis. No PE occurred. Female patients and patients undergoing TKA had higher incidence of postoperative VTE. Patients who developed postoperative VTE were older, and had higher levels of preoperative D-Dimer and Caprini score. A high level of preoperative D-dimer (OR = 2.11, 95 %CI = 1.35–3.30) and the surgery of TKA (OR = 2.29, 95 %CI = 1.29–4.01) significantly increased the risk of developing postoperative VTE. Postoperative initiation of concomitant mechanical prophylaxis and low molecular weight heparin (LMWH) was protective for postoperative VTE (OR = 0.56, 95 %CI = 0.37–0.86).

**Conclusions:**

VTE is common in patients with diabetes undergoing joint arthroplasty. Patients undergoing TKA or with a high level of preoperative D-dimer are at a considerable risk of developing postoperative VTE. There may be a protective role of postoperative initiation of concomitant mechanical prophylaxis and LMWH for VTE.

## Background

The aging population and rising incidence of arthritis have caused an increasing number of people undergoing joint arthroplasty, such as total hip arthroplasty (THA) or total knee arthroplasty (TKA). Over the last several decades, the global prevalence of diabetes has also increased significantly [1]. In China, there are currently more than 110 million people with diabetes and about 20 % of people aged over 60 years old have diabetes[1]. Therefore, the number of people with diabetes selecting to undergo joint arthroplasty is proportionally increasing [2, 3]. In 2008, a large study from the U.S. including 751,340 THA or TKA patients revealed that 8.55 % of patients have the comorbidity of diabetes, and the risk of postoperative complications increased significantly for patients with diabetes [4].

Venous thromboembolism (VTE), including deep venous thrombosis (DVT) and pulmonary embolism (PE), is a common postoperative complication of arthroplasty, leading to an increase of mortality and morbidity, as well as an increase of the cost of care [5]. The incidence of VTE following orthopedic surgery is highly variable due to heterogeneities of the studied populations, different treatment strategies and diagnostic measures, with published rates ranging from 1–2% to 60 % [6]. VTE is the formation of a blood clot inside a vein that obstructs the normal flow of blood, and may be precipitated by three factors, venous stasis, blood hypercoagulability and endothelial dysfunction. Evidence shows that diabetes is related to a few changes in coagulation and fibrinolysis that may lead to thrombophilia [7]. A longitudinal study investigating relationship between risk factors of coronary heart disease and VTE reported that diabetes and obesity both significantly increased the risk of incident VTE independently of age, race and sex [8]. Those with diabetes at baseline had a 1.7-fold greater risk for VTE than those with normal fasting glucose levels [8]. Additionally, patients with diabetes who subsequently developed VTE had a 74 % increase in the risk of recurrent DVT and 40 % increase in the risk of long-term major bleeding [9].

Although guidelines recommend routine VTE prophylaxis after arthroplasty surgery [10], the appropriate strategy for preventing VTE in high risk patients has been debated over several decades. VTE prophylaxis is apparently more impotant for patients with diabetes undergoing THA or TKA, while pharmacologic prophylaxis presents risks including heparin-induced thrombocytopenia and bleeding complications. In order to balance benefits and adverse effects of VTE prophylaxis, various risk assessment models (RAMs) for VTE have been developed in Western countries to stratify patients based on risk factors and help to deliver individualized VTE prophylaxis [11–13]. However, risk factors derived from certain populations may not be applicable to other ethnic groups. Moreover, almost all patients with diabetes following THA or TKA are classified as high risk group of developing VTE by these RAMs; therefore, specific attention should be paid to this population. To our knowledge, there is no previous study to assess the risk factors of VTE specific to Chinese patients with diabetes following THA or TKA. The aim of our study was to present the incidence of VTE among people with diabetes following THA or TKA and investigate its associated risk factors in current clinical practice. Such information is important for the prediction of postoperative VTE and can be incorporated into the choice of appropriate VTE prophylaxis in this high risk population.

## Methods

### Data sources

**Fig. 1:**
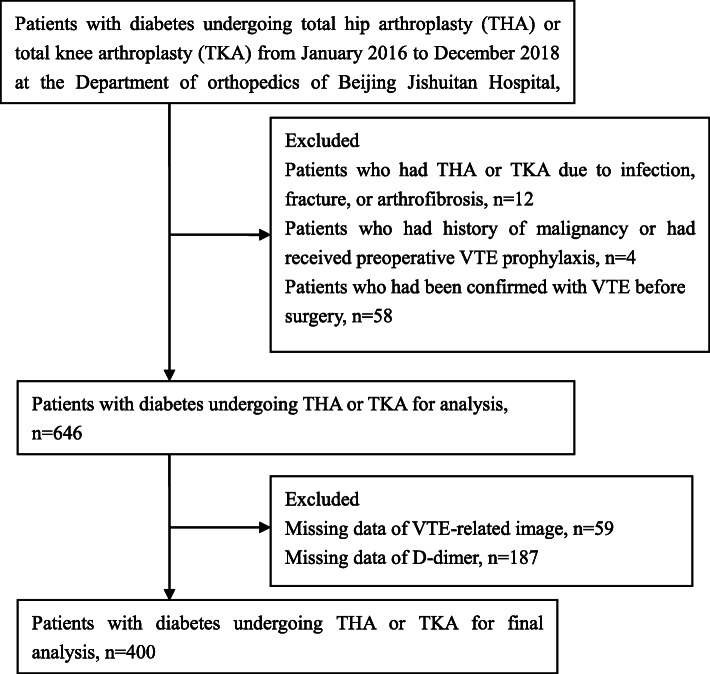
Detailed approach for the selection of participants

From January 2016 to December 2018, 720 patients with diabetes had undergone THA or TKA at the Department of orthopedics in Beijing Jishuitan Hospital. We excluded 12 patients who had THA or TKA due to infection, fracture, or arthrofibrosis; 4 patients who had history of malignancy or had received preoperative VTE prophylaxis; 58 patients who had been diagonsed with VTE before surgery; 246 patients without VTE-related images or tests of pre-operative D-dimer, leaving 400 patients for analyses (Fig. 1). The demographic and clinical information was obtained from electronic medical records.

### Definition of VTE and diabetes

Trained and certified sonographers performed lower limb venous Doppler ultrasound on patients before and 3 to 5 days after surgery for screening of DVT using a standardized method.

Computer tomography pulmonary angiography (CTPA) was done to confirm PE for those with new postoperative DVT and typical symptoms of PE. A VTE event was determined to have occurred if either a DVT or PE as defined previously occurred. Distal vein thrombosis is defined as the presence of venous thrombus below the popliteal vein, including posterior tibial vein, peroneal vein, anterior tibial vein, and muscular calf vein (soleus or gastrocnemius vein). Proximal vein thrombosis is defined as the presence of venous thrombus in popliteal veins or above.

Patients were considered to have prevalent diabetes when this was documented in medical records or taking antiglycaemic agents for previously diagnosed diabetes.

### Other demographic and clinical measures

The clinical status was evaluated when patients were admitted. Detailed anthropometric measurements were collected by trained nurses, adhering to standardized techniques. Standing height was measured to an accuracy of 0.1 cm and weight to an accuracy of 0.1 kg. The body mass index (BMI) was calculated as weight in kilogram divided by the square of the height in meters. Blood pressure was measured twice on the right arm with an electronic sphygmomanometer (HEM-907; Omron Healthcare Company, Kyoto, Japan), in the sitting position, and was rounded to the nearest 1 mmHg. The average readings were used. Venous blood samples were obtained by nurses after an overnight fasting for 8-10 h. Fasting levels of blood glucose were measured using an autoanalyzer (Hitachi 7600-110E/ Hitachi 7180E; Hitachi, Tokyo, Japan). Fasting levels of D-dimer were measured by immunoturbidimetric assay using an automatic coagulation analyzer (Sysmex CS-5100, Sysmex Corporation, Japan). HbA1c concentration was measured by high-pressure liquid chromatography (D-10 Hemoglobin Testing System, Bio-Rad Laboratories, Inc., Schiltigheim, France).

Information about postoperative prophylaxis regimens was collected from electronic medical records. Mechanical prophylaxis was defined as either elastic stockings or foot pump. IRB Approval (JST201609-08) was obtained from the Ethics Committees of Beijing Jishuitan Hospital and written informed consent was obtained from all participants.

### Statistical analysis

Data analysis was carried out using SPSS version 22.0 (SPSS Inc., Chicago, IL, USA). Data were presented as median (25th, 75th Quartile), mean ± SD or n (%). Continuous variables were compared using either student’s t test or Mann- Whitney U test. Categorical variables were compared using χ2 test or Fisher’s exact test. Age (≥ 65 years versus < 65 years), sex (female versus male), smoking (smoking versus no smoking), preoperative comorbidities (hypertension, cerebrovascular disease and varicose veins of lower extremity versus none), BMI (≥ 28 kg/m^2^ versus < 28 kg/m^2^), SBP (≥ 140mmHg versus < 140mmHg ), DBP (≥ 90mmHg versus < 90mmHg ), operative procedures (TKA versus THA), HbA_1_c (≥ 7.5 % versus < 7.5 %), preoperative D-dimer (> 0.55 mg/L FEU versus ≤ 0.55 mg/L FEU) and postoperative prophylaxis regimens (mechanical prophylaxis versus concomitant mechanical prophylaxis and LMWH) were treated as independent variables, which were previously shown to be risk factors for VTE. A multivariable logistic regression analysis was performed to determine the independent risk factors for VTE. All analyses were two-tailed and *P* < 0.05 was considered to be statistically significant.

## Results

The detailed approach for participants’ selection is shown in Fig. 1. A total of 400 patients with diabetes who underwent THA or TKA between January 2016 and December 2018 at Beijing Jishuitan Hospital were included in this study, 24.3 % of whom were males. The mean age of these patients was 64.9 years. The overall incidence of postoperative VTE in this population was 46.8 % (187 of 400). Among 187 VTE patients, 7.5 % (14 out of 187) had proximal vein thrombosis and 92.5 % had distal vein thrombosis. No PE occurred. The incidence of VTE in patients who received THA was 32.3 % (30 out of 97) with 3.3 % of proximal vein thrombosis (1 out of 30), while the incidence of VTE after TKA was 51.1 % (157 out of 307) with 8.3 % of proximal vein thrombosis (13 out of 157).

Demographic and clinical characteristics of patients by VTE status are presented in Table 1. There were more females who developed VTE in this population. The mean surgery age of patients who developed VTE was significantly older than those without VTE (66.0 versus 64.1 years, *P* = 0.012). Furthermore, the preoperative D-dimer in VTE patients was higher than that in those without VTE (0.46 versus 0.40 mg/L FEU, *P* = 0.007). There was no difference in terms of prevalence of smoking, hypertension, cerebrovascular disease or varicose veins, BMI, SBP, DBP, preoperative fasting glucose, HbA1c and Caprini score between patients with and without VTE. Among this population, 55.5 % of patients were given mechanical prophylaxis after THA or TKA for VTE prophylaxis, while 45.5 % of patients were given concomitant mechanical prophylaxis and low molecular weight heparin (LMWH). VTE incidence in patients who were given concomitant mechanical prophylaxis and LMWH after THA or TKA was significantly lower than that in those who were given mechanical prophylaxis alone (Table 2).

**Table 1:** Demographic and clinical characteristics of patients with diabetes undergoing total hip arthroplasty (THA) or total knee arthroplasty (TKA)

	Total	VTE (+)	VTE (-)	Z value/t value/χ2 value	*P* value
Sex (M/F)	97/303	34/153	63/150	7.040	0.010
Age (years)	64.9 ± 7.7	66.0 ± 6.7	64.1 ± 8.3	-2.513	0.012
Smoke, % (n)	16.5 (66)	13.9 (26)	18.8 (40)	1.718	0.225
Alcohol drink, % (n)	11.5 (46)	11.2 (21)	11.7 (25)	0.025	1.000
Hypertension, % (n)	64.8 (259)	64.7 (121)	64.8 (138)	0.000	1.000
Cerebrovascular disease, % (n)	14.0 (56)	15.0 (28)	13.1 (28)	0.276	0.665
BMI (kg/m^2^)	26.9 ± 3.4	27.1 ± 3.5	26.7 ± 3.4	-1.225	0.221
SBP (mmHg)	144 (131, 157)	144 (133, 157)	143 (130, 157)	-1.001	0.317
DBP (mmHg)	80 (73, 88)	80 (72, 88)	80 (73, 88)	-0.351	0.725
Preoperative fasting glucose (mmol/L)	7.7 ± 2.2	7.8 ± 2.3	7.7 ± 2.1	0.104	0.917
HbA1c (%)	7.5 ± 1.5	7.6 ± 1.6	7.5 ± 1.4	0.189	0.835
Preoperative D-dimer (mg/L FEU)	0.43 (0.27, 0.83)	0.46 (0.28, 1.14)	0.40 (0.27, 0.66)	-2.696	0.007
Varicose veins, % (n)	8.3 (33)	8.0 (15)	8.5 (18)	0.024	1.000
Operative procedures					
THA, % (n)	23.3 (93)	32.3 (30)	67.7 (63)	10.222	0.001
TKA, % (n)	76.8 (307)	51.1 (157)	48.9 (150)		
Caprini score	7.2 ± 0.7	7.2 ± 0.8	7.1 ± 0.8	-0.827	0.409

**Table 2:** Postoperative prophylaxis regimens of patients with diabetes undergoing total joint arthroplasty

	Total	VTE (+)	VTE (-)	Z value/t value/χ2 value	*P* value
Mechanical prophylaxis, % (n)	55.5 (222)	29.5 (118)	26.0 (104)	8.216	0.005
Mechanical prophylaxis + Low molecular weight heparin, % (n)	44.5 (178)	17.3 (69)	27.2 (109)		

Table 3 summarises the results of the multivariable logistic regression analysis for postoperative VTE. We found that TKA was associated with 2.3-fold higher risk of VTE as compared to THA. Preoperative D-dimer > 0.55 (mg/L FEU) was associated with 2.1-fold higher risk of VTE as compared to preoperative D-dimer ≤ 0.55 (mg/L FEU). Mechanical prophylaxis and LMWH combined reduced the risk of VTE by 44 % as compared with mechanical prophylaxis alone.

**Table 3:** Adjusted odds ratios and 95 % CI between risk factors and VTE status

Risk factors	OR	95 % CI for OR	*P* value
Age (≥ 65 years versus < 65 years)	0.904	0.585–1.399	0.652
Sex (female versus male)	1.541	0.862–2.755	0.144
Smoking (yes versus no)	1.234	0.633–2.405	0.537
Varicose veins (yes versus no)	0.769	0.367–1.729	0.565
Hypertension (yes versus no)	0.828	0.518–1.326	0.432
Cerebrovascular disease (yes versus no)	1.136	0.616–2.094	0.683
BMI (≥ 28 kg/m^2^ versus < 28 kg/m^2^)	0.834	0.536-1.300	0.423
SBP (≥ 140mmHg versus < 140mmHg )	1.143	0.720–1.812	0.571
DBP (≥ 90mmHg versus < 90mmHg )	0.976	0.561–1.699	0.932
Operative procedures (TKA versus THA)	2.285	1.287–4.058	0.005
HbA_1_c (≥ 7.5 % versus < 7.5 %)	1.232	0.766–1.747	0.273
Preoperative D-dimer (> 0.55 mg/L FEU versus ≤ 0.55 mg/L FEU)	2.109	1.348-3.300	0.001
Postoperative prophylaxis regimens (mechanical prophylaxis versus concomitant mechanical prophylaxis and LMWH)	0.559	0.365–0.855	0.007

## Discussion

The overall incidence of postoperative VTE was 46.8 % in patients with diabetes undergoing THA or TKA in this single center study and no PEs were confirmed. The majority of postoperative DVT identified in this study was distal vein thrombosis, which represented over 90 % of all lower limbs DVT. Patients with diabetes undergoing joint arthroplasty remain at high risk for developing postoperative VTE. Since guidelines for VTE prophylaxis were published, multiple strategies for VTE prophylaxis have been routinely applied to most patients after arthroplasty. To our knowledge, this study is the first to investigate the incidence of VTE and associated risk factors among patients with diabetes following joint arthroplasty under the real-world clinical practice, which may help to guide orthopaedic surgeons in their choice of VTE prophylaxis. We found that the VTE incidence for those undergoing TKA was 51.1 %, which was higher than those undergoing THA. TKA was associated with 2.3-fold higher risk of postoperative VTE as compared with THA. This is consistent with other studies [14, 15]. It is possibly due to more extensive damage to soft-tissue and bone during TKA, which leads to the local release of tissue factors, thus initiating the coagulation cascade, and also destructs the vascular anatomy [16].

D-dimer is a degradation product of cross-linked fibrin, and therefore a biomarker of coagulation activation and fibrinolysis. Plasma D-dimer level is often measured in order to screen VTE. A low D-dimer level may be useful for excluding acute thrombosis in clinical settings. However, there is a paucity of studies with respect to D-dimer as a risk factor for incident VTE. Our study suggested that high level of preoperative D-dimer was an independent risk factor for postoperative VTE. This is supported by the findings of Cushman et al. [17], who demonstrated that higher baseline D-dimer level was correlated with increased risk of subsequent VTE using a sample of the general US population. Another two case-control studies showed that patients with a history of VTE were more likely to have elevated D-dimer level than controls [18, 19]. A potential explanation proposed for the association between D-dimer and VTE is that D-dimer may be a marker for other factors associated with the pathophysiology of VTE. Evidence indicates that D-dimer is higher in the presence of genetic risk factors (e.g. prothrombin 20,210 A, factor V Leiden, or elevated factor VIII:c) for VTE [20]. However, these genetic mutations are rare in Asian populations [21]. In addition, some possible risk factors for VTE such as age, smoking and inflammatory status are also reported to be associated with higher D-dimer [22–24]. Therefore, measurement of baseline D-dimer may provide comprehensive clinical information than assessment of some specific thrombosis risk factors, such as genes or obesity.

Given that VTE prophylaxis has become the standard of care for patients undergoing THA or TKA, early mobilization combined with mechanical compressive device is highly recommended and has been the primary method of VTE prophylaxis in our center since 2012. In our study, all patients used mechanical prophylaxis for VTE after surgery and less than half of patients used LMWH. Concomitant use of mechanical prophylaxis and LMWH decreased the risk of VTE by 43 % as compared with mechanical prophylaxis alone. A meta-analysis including 14 trials showed that LMWH decreased asymptomatic DVT by 50 % (combined risk ratio [RR], 0.50; 95 % CI, 0.43–0.59) for major orthopedic surgery without significant increase in bleeding rates, which is similar with our result [10]. However, it is noteworthy to weigh the bleeding risk versus thrombotic risk before commencing pharmacological prophylaxis, especially for situations where the patients’ personal bleeding risks are high.

There are various RAMs for VTE in Western countries, among which Caprini RAM is often applied to orthopedics inpatients. THA or TKA would give a score of 5 if assessed by the Caprini RAM. This places all patients in the high-risk category. In our study, no significant difference was found about Caprini score between patients with and without VTE. Therefore, Caprini RAM is highly sensitive but poorly specific for patients undergoing THA or TKA, which might not facilitate managing diverse patients with personalized VTE prophylatic strategies. Further studies need to be carried out to develop a specific VTE RAM that serves this high risk population.

This study has several limitations. First, this study was carried out in only one large center, which limits its external validity in other population. Second, VTE in our study is defined as all venous thromboembolism including distal vein thrombosis, proximal vein thrombosis and PE. Whether distal vein thrombosis requires anticoagulant therapy is currently a debated issue due to its uncertain clinical significance. However, some investigators reported that therapeutic anticoagulation was associated with a significant reduction in the risk of extension to proximal vein thrombosis or PE among patients with distal vein thrombosis [25, 26]. Current American College of Chest Physicians guideline recommends therapertic anticoagulation for distal vein thrombosis under the presence of the risk factors, such as active cancer, history of VTE or inpatient status [10]. As patients enrolled in our study could all be classified at high risk of developing VTE based on Caprini RAM, it might be reasonable to include distal vein thrombosis in the analysis. Third, our study only investigated in-hospital VTE. With some VTEs occurring post-discharge, within 30–45 days of surgery, our study may underestimate the true incidence of VTEs in the postoperative period. Additionally, although ultrasonography is a sensitive method for the detection of DVTs [27], it’s an operator-dependent technique [28]. We didn’t appoint the same sonographer to perform the test, which might present an inter-operator variability. However, Razek AA et al. reported that the inter-operator agreement of color duplex ultrasound for detection of complete occlusion with thrombosis was excellent with Kappa coefficient value of 0.84 [29]. Lastly, lack of investigation of other risk factors such as anesthesia type, perioperative red blood cell transfusion and plasma homocysteine was also a limitation of this study.

## Conclusions

VTE is common in patients with diabetes following joint arthroplasty. Patients undergoing TKA or with a high level of preoperative D-dimer are at an increasing risk of VTE. There may be a protective role of postoperative initiation of concomitant mechanical prophylaxis and LMWH for VTE.

## Data Availability

The datasets during and/or analysed during the current study are available from the corresponding author on reasonable request.
